# Vieillardiixanthone B^1^

**DOI:** 10.1107/S1600536810007026

**Published:** 2010-03-13

**Authors:** Nawong Boonnak, Suchada Chantrapromma, Hoong-Kun Fun, Chatchanok Karalai

**Affiliations:** a Crystal Materials Research Unit, Department of Chemistry, Faculty of Science, Prince of Songkla University, Hat-Yai, Songkhla 90112, Thailand; b X-ray Crystallography Unit, School of Physics, Universiti Sains Malaysia, 11800 USM, Penang, Malaysia

## Abstract

The title compound [systematic name: 1,5-dihydr­oxy-3,6-dimeth­oxy-4-(2-methyl­but-3-en-2-yl)-9*H*-xanthen-9-one], C_20_H_20_O_6_, is a xanthone, which was isolated from the roots of *Cratoxylum formosum* ssp. *pruniflorum*. The three rings in the mol­ecule are approximately coplanar, with an r.m.s. deviation of 0.0372 (2) Å for the plane through the 14 non-H atoms. The O atoms of the two hydr­oxy substituents also lie close to this plane with deviations of 0.0669 (2) and 0.1122 (2) Å, respectively. The 1,1-dimethyl-2-propenyl substituent is in a (−)-anti­clinal conformation. Intra­molecular O—H⋯O hydrogen bonds generate *S*(5) and *S*(6) ring motifs. In the crystal, mol­ecules are linked into infinite chains along [010] by O—H⋯O hydrogen bonds and weak C—H⋯O inter­actions. π–π inter­actions with centroid–centroid distances of 3.6172 (10) and 3.6815 (10) Å are also observed.

## Related literature

For hydrogen-bond motifs, see: Bernstein *et al.* (1995 ▶). For bond-length data, see: Allen *et al.* (1987 ▶). For background to xanthones and their biological activity, see: Boonnak, Karalai *et al.* (2006 ▶, 2007 ▶, 2009 ▶); Hay *et al.* (2008 ▶). For a related structure, see: Boonnak, Chantrapromma & Fun (2006 ▶). For the stability of the temperature controller used in the data collection, see Cosier & Glazer, (1986 ▶).
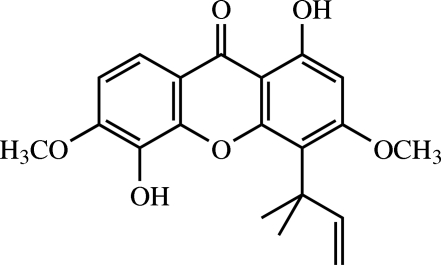


## Experimental

### 

#### Crystal data

C_20_H_20_O_6_
*M* *_r_* = 356.36
Monoclinic, 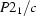

*a* = 12.1500 (4) Å
*b* = 14.7396 (4) Å
*c* = 9.5177 (3) Å
β = 90.208 (2)°
*V* = 1704.48 (9) Å^3^
*Z* = 4
Mo *K*α radiation
μ = 0.10 mm^−1^
*T* = 100 K
0.50 × 0.23 × 0.22 mm

#### Data collection

Bruker APEXII CCD area-detector diffractometer
Absorption correction: multi-scan (*SADABS*; Bruker, 2005 ▶) *T*_min_ = 0.951, *T*_max_ = 0.978
37868 measured reflections
3906 independent reflections
2682 reflections with *I* > 2σ(*I*)
*R*_int_ = 0.071

#### Refinement

*R*[*F* ^2^ > 2σ(*F* ^2^)] = 0.054
*wR*(*F* ^2^) = 0.133
*S* = 1.03
3906 reflections
259 parameters
H atoms treated by a mixture of independent and constrained refinement
Δρ_max_ = 0.27 e Å^−3^
Δρ_min_ = −0.27 e Å^−3^

### 

Data collection: *APEX2* (Bruker, 2005 ▶); cell refinement: *SAINT* (Bruker, 2005 ▶); data reduction: *SAINT*; program(s) used to solve structure: *SHELXTL* (Sheldrick, 2008 ▶); program(s) used to refine structure: *SHELXTL*; molecular graphics: *SHELXTL*; software used to prepare material for publication: *SHELXTL* and *PLATON* (Spek, 2009 ▶).

## Supplementary Material

Crystal structure: contains datablocks global, I. DOI: 10.1107/S1600536810007026/sj2732sup1.cif

Structure factors: contains datablocks I. DOI: 10.1107/S1600536810007026/sj2732Isup2.hkl

Additional supplementary materials:  crystallographic information; 3D view; checkCIF report

## Figures and Tables

**Table 1:** Hydrogen-bond geometry (Å, °)

*D*—H⋯*A*	*D*—H	H⋯*A*	*D*⋯*A*	*D*—H⋯*A*
O3—H1*O*3⋯O2	0.95 (3)	1.65 (3)	2.5573 (18)	160 (2)
O5—H1*O*5⋯O6	0.83 (2)	2.25 (2)	2.7019 (19)	115 (2)
O5—H1*O*5⋯O2^i^	0.83 (2)	1.98 (2)	2.7520 (18)	155 (2)
C8—H8*A*⋯O5^ii^	0.93	2.54	3.413 (2)	157

Symmetry codes: (i) 
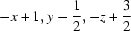
; (ii) 
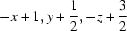
.

**Fig. 1.**
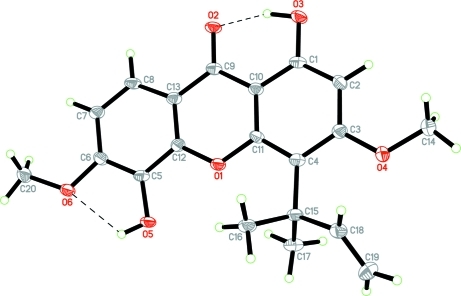
The structure of (I), showing 50% probability displacement ellipsoids and the atom-numbering scheme. Intramolecular hydrogen bonds are shown as dashed lines.

**Fig. 2.**
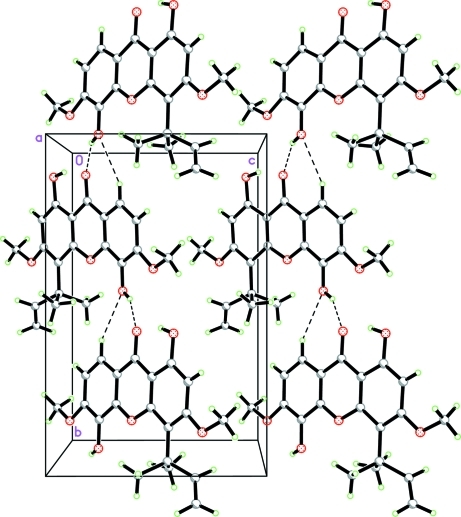
The crystal packing of (I) viewed along the a axis, showing one dimensional chains along the [010] direction. Hydrogen bonds are shown as dashed lines.

## supplementary crystallographic information

### Comment

During the course of our studies into the chemical constituents and bioactive compounds from Thai medicinal plants, we previously reported the isolation of xanthones from the *Cratoxylum formosum* ssp. *pruniflorum*. We found that several isolated xanthones showed antibacterial, antifungal and cytotoxic activities (Boonnak, Karalai *et al.*, 2006; 2007; 2009). Further isolation of materials from the roots of this plant resulted in the title xanthone known as vieillardiixanthone B (Hay *et al.*, 2008). It was tested against both Gram-positive and Gram-negative bacteria i.e. *Bacillus subtilis*, *Staphylococcus aureus* TISTR517, *Enterococcus faecalis* TISTR459, Methicillin-Resistant *Staphylococcus aureus* (MRSA) ATCC43300, Vancomycin-Risistant *Enterococcus faecalis* (VRE) ATCC 51299, *Streptococcus faecalis*, *Salmonella typhi*, *Shigella sonei* and *Pseudomonas aeruginosa*. Our results showed that the title compound has no antibacterial action against these pathogens. Herein we report the crystal structure of the title xanthone (I).

In compound (I) (Fig. 1), the three ring system [C1–C13/O1] is essentially planar with an r.m.s. deviation of 0.0372 (2) Å from the plane through all non-hydrogen atoms of the three rings and with a maximum deviation of -0.100 (2) Å for atom C4. The O3 and O5 hydroxyl O atoms lie close to this plane with deviations +0.0669 (2) for O3 and +0.1122 (2) Å for O5. The two methoxy groups lie close to the planes of their benzene rings with torsion angles C14–O4–C3–C2 = 3.8 (3)° and C20–O6–C6–C7 = -7.4 (3)°. The 1,1-dimethyl-2-propenyl [C15–C19] substituent is in a (-)-anticlinal conformation as indicated by the torsion angle C4–C15–C18–C19 = -132.1 (2)°. Intramolecular O3—H1O3···O2 and O5—H1O5..O6 hydrogen bonds (Table 1) generate S(6) and S(5) ring motifs, respectively (Bernstein *et al.*, 1995). The bond distances in (I) are within normal ranges (Allen *et al.*, 1987) and comparable to those in a related structure (Boonnak, Chantrapromma & Fun, 2006).

The crystal packing of (I) is stabilized by intermolecular O—H···O hydrogen bonds and weak C—H···O interactions (Table 1). The molecules are linked into infinite one dimensional chains along [010] by O—H···O and C—H···O hydrogen bonds (Fig. 2 and Table 1). π–π interactions with distances Cg_1_···Cg_2_ = 3.6172 (10) Å (symmetry code: x, 1/2-y, z) and Cg_1_···Cg_3_ = 3.6815 (10) Å (symmetry code: x, 1/2-y, -1/2+z) were also observed; Cg_1_, Cg_2_ and Cg_3_ are the centroids of O1/C9–C13, C1–C4/C10–C11 and C5–C8/C12–C13 rings, respectively.

### Experimental

The air-dried roots of *C. formosum* ssp. *pruniflorum* (5.00 kg) was extracted with CH_2_Cl_2_ (2 x 20 L, for a week) at room temperature and was further evaporated under reduced pressure to afford a deep green crude CH_2_Cl_2_ extract (58.87 g), which was subjected to QCC (Quick Column Chromatography) on silica gel using n-hexane as a first eluent and then increasing the polarity with acetone to give 12 fractions (F1-F12). Fractions F8-F11 were combined and separated by QCC eluting with 30% EtOAc-n-hexane to give 8 subfractions (F8A-F8H). Subfractions F8E and F8F were combined and separated by QCC and eluted with 30% EtOAc-n-hexane to obtain 20 subfractions (F8E1-F8E20). Subfractions F8E10-F8E12 were combined and separated by QCC and eluted with a gradient of CH_2_Cl_2_-n-hexane to give 12 subfractions (F8E10A-F8E10L). Subfraction F8E10D was separated by CC (Column Chromatography) eluting with 10% acetone-n-hexane to give 8 subfractions (F8E10D1-F8E10D8). Subfraction F8E10D5 was further purified by CC and eluted with a gradient of CH_2_Cl_2_-n-hexane to give the title compound as a yellow solid (3.5 mg). Yellow block-shaped single crystals of the title compound suitable for *x*-ray structure determination were recrystallized from acetone/CH_3_OH (9.5:0.5, v/v) after several days (M.p. 486-488 K).

### Refinement

Hydroxy H atoms attached to O3 and O5 and H atoms attached to C18 and C19 were located from the difference map and refined isotropically. The remaining H atoms were placed in calculated positions with d(C—H) = 0.93 Å for aromatic and 0.96 Å for CH_3_ atoms. The *U*_iso_ values were constrained to be 1.5*U*_eq_ of the carrier atom for methyl H atoms and 1.2*U*_eq_ for the remaining H atoms. A rotating group model was used for the methyl groups. The highest residual electron density peak is located at 0.72 Å from C15 and the deepest hole is located at 0.72 Å from C5.

### Crystal data

**Table:**

C_20_H_20_O_6_	*F*(000) = 752
*M**_r_* = 356.36	*D*_x_ = 1.389 Mg m^−^^3^
Monoclinic, *P*2_1_/*c*	Melting point = 486–488 K
Hall symbol: -P 2ybc	Mo *K*α radiation, λ = 0.71073 Å
*a* = 12.1500 (4) Å	Cell parameters from 3906 reflections
*b* = 14.7396 (4) Å	θ = 2.2–27.5°
*c* = 9.5177 (3) Å	µ = 0.10 mm^−^^1^
β = 90.208 (2)°	*T* = 100 K
*V* = 1704.48 (9) Å^3^	Block, yellow
*Z* = 4	0.50 × 0.23 × 0.22 mm

### Data collection

**Table:**

Bruker APEXII CCD area-detector diffractometer	3906 independent reflections
Radiation source: sealed tube	2682 reflections with *I* > 2σ(*I*)
graphite	*R*_int_ = 0.071
φ and ω scans	θ_max_ = 27.5°, θ_min_ = 2.2°
Absorption correction: multi-scan (*SADABS*; Bruker, 2005)	*h* = −15→15
*T*_min_ = 0.951, *T*_max_ = 0.978	*k* = −19→19
37868 measured reflections	*l* = −12→12

### Refinement

**Table:**

Refinement on *F*^2^	Primary atom site location: structure-invariant direct methods
Least-squares matrix: full	Secondary atom site location: difference Fourier map
*R*[*F*^2^ > 2σ(*F*^2^)] = 0.054	Hydrogen site location: inferred from neighbouring sites
*wR*(*F*^2^) = 0.133	H atoms treated by a mixture of independent and constrained refinement
*S* = 1.03	*w* = 1/[σ^2^(*F*_o_^2^) + (0.0609*P*)^2^ + 0.5536*P*] where *P* = (*F*_o_^2^ + 2*F*_c_^2^)/3
3906 reflections	(Δ/σ)_max_ < 0.001
259 parameters	Δρ_max_ = 0.27 e Å^−^^3^
0 restraints	Δρ_min_ = −0.27 e Å^−^^3^

### Special details

**Table:**

Experimental. The crystal was placed in the cold stream of an Oxford Cryosystems Cobra open-flow nitrogen cryostat (Cosier & Glazer, 1986) operating at 100.0 (1) K.
Geometry. All esds (except the esd in the dihedral angle between two l.s. planes) are estimated using the full covariance matrix. The cell esds are taken into account individually in the estimation of esds in distances, angles and torsion angles; correlations between esds in cell parameters are only used when they are defined by crystal symmetry. An approximate (isotropic) treatment of cell esds is used for estimating esds involving l.s. planes.
Refinement. Refinement of F^2^ against ALL reflections. The weighted R-factor wR and goodness of fit S are based on F^2^, conventional R-factors R are based on F, with F set to zero for negative F^2^. The threshold expression of F^2^ > 2sigma(F^2^) is used only for calculating R-factors(gt) etc. and is not relevant to the choice of reflections for refinement. R-factors based on F^2^ are statistically about twice as large as those based on F, and R- factors based on ALL data will be even larger.

### Fractional atomic coordinates and isotropic or equivalent isotropic displacement parameters (Å^2^)

**Table:**

	*x*	*y*	*z*	*U*_iso_*/*U*_eq_	
O1	0.35090 (10)	0.13014 (8)	0.63535 (13)	0.0187 (3)	
O2	0.37354 (10)	0.40673 (8)	0.61270 (13)	0.0207 (3)	
O3	0.21931 (11)	0.40714 (8)	0.42914 (14)	0.0230 (3)	
H1O3	0.278 (2)	0.4208 (18)	0.491 (3)	0.055 (8)*	
O4	0.07417 (11)	0.12110 (9)	0.29203 (14)	0.0257 (3)	
O5	0.46803 (11)	0.02998 (8)	0.80738 (14)	0.0202 (3)	
H1O5	0.522 (2)	0.0062 (16)	0.847 (2)	0.038 (7)*	
O6	0.62914 (11)	0.10303 (8)	0.96779 (14)	0.0222 (3)	
C1	0.21969 (15)	0.31542 (12)	0.43964 (19)	0.0186 (4)	
C2	0.14722 (15)	0.26604 (12)	0.35834 (19)	0.0195 (4)	
H2A	0.0986	0.2956	0.2981	0.023*	
C3	0.14717 (15)	0.17086 (12)	0.36683 (19)	0.0187 (4)	
C4	0.22012 (14)	0.12173 (12)	0.45343 (19)	0.0176 (4)	
C5	0.48867 (15)	0.12058 (11)	0.80499 (19)	0.0174 (4)	
C6	0.56984 (15)	0.16161 (12)	0.88664 (19)	0.0188 (4)	
C7	0.58547 (16)	0.25558 (12)	0.8834 (2)	0.0201 (4)	
H7A	0.6400	0.2822	0.9385	0.024*	
C8	0.51960 (15)	0.30887 (12)	0.79793 (19)	0.0202 (4)	
H8A	0.5291	0.3715	0.7972	0.024*	
C9	0.36821 (15)	0.32157 (11)	0.62009 (18)	0.0171 (4)	
C10	0.29140 (14)	0.27097 (11)	0.53441 (19)	0.0168 (4)	
C11	0.28714 (14)	0.17552 (12)	0.53963 (19)	0.0166 (4)	
C12	0.42547 (14)	0.17531 (12)	0.71676 (18)	0.0166 (4)	
C13	0.43899 (15)	0.26940 (11)	0.71284 (19)	0.0174 (4)	
C14	0.00024 (16)	0.16425 (14)	0.1967 (2)	0.0265 (5)	
H14A	−0.0397	0.1190	0.1449	0.040*	
H14B	0.0411	0.2015	0.1328	0.040*	
H14C	−0.0505	0.2013	0.2483	0.040*	
C15	0.22616 (15)	0.01618 (12)	0.45818 (19)	0.0184 (4)	
C16	0.34762 (16)	−0.01610 (12)	0.4569 (2)	0.0221 (4)	
H16A	0.3505	−0.0789	0.4305	0.033*	
H16B	0.3791	−0.0087	0.5488	0.033*	
H16C	0.3885	0.0193	0.3904	0.033*	
C17	0.16997 (17)	−0.01792 (12)	0.5923 (2)	0.0250 (4)	
H17A	0.0927	−0.0044	0.5879	0.038*	
H17B	0.2020	0.0116	0.6726	0.038*	
H17C	0.1801	−0.0823	0.6004	0.038*	
C18	0.17864 (16)	−0.02757 (12)	0.3267 (2)	0.0223 (4)	
H18	0.2133 (17)	−0.0069 (14)	0.240 (2)	0.028 (6)*	
C19	0.10821 (18)	−0.09584 (14)	0.3216 (3)	0.0316 (5)	
H19B	0.0844 (17)	−0.1203 (14)	0.231 (2)	0.030 (6)*	
H19A	0.0703 (18)	−0.1196 (15)	0.407 (2)	0.038 (6)*	
C20	0.72165 (16)	0.13735 (13)	1.0432 (2)	0.0256 (5)	
H20A	0.7565	0.0888	1.0937	0.038*	
H20B	0.6977	0.1830	1.1082	0.038*	
H20C	0.7731	0.1634	0.9784	0.038*	

### Atomic displacement parameters (Å^2^)

**Table:**

	*U*^11^	*U*^22^	*U*^33^	*U*^12^	*U*^13^	*U*^23^
O1	0.0254 (7)	0.0110 (6)	0.0195 (7)	−0.0006 (5)	−0.0059 (6)	0.0008 (5)
O2	0.0294 (7)	0.0091 (6)	0.0236 (7)	−0.0007 (5)	−0.0048 (6)	0.0011 (5)
O3	0.0304 (8)	0.0116 (6)	0.0270 (8)	0.0018 (5)	−0.0076 (6)	0.0025 (6)
O4	0.0277 (8)	0.0176 (7)	0.0317 (8)	−0.0013 (5)	−0.0153 (6)	0.0021 (6)
O5	0.0266 (8)	0.0089 (6)	0.0251 (7)	−0.0005 (5)	−0.0072 (6)	0.0011 (5)
O6	0.0281 (7)	0.0123 (6)	0.0259 (7)	−0.0004 (5)	−0.0115 (6)	0.0017 (5)
C1	0.0246 (10)	0.0113 (8)	0.0199 (10)	0.0003 (7)	0.0024 (8)	0.0016 (7)
C2	0.0219 (10)	0.0171 (9)	0.0195 (10)	0.0023 (7)	−0.0041 (8)	0.0029 (7)
C3	0.0220 (10)	0.0165 (9)	0.0177 (9)	−0.0014 (7)	−0.0019 (8)	0.0002 (7)
C4	0.0214 (10)	0.0131 (8)	0.0183 (9)	0.0001 (7)	0.0005 (8)	0.0013 (7)
C5	0.0233 (10)	0.0100 (8)	0.0188 (9)	0.0000 (7)	0.0004 (8)	−0.0003 (7)
C6	0.0229 (10)	0.0150 (9)	0.0185 (10)	0.0016 (7)	−0.0042 (8)	0.0008 (7)
C7	0.0254 (10)	0.0130 (9)	0.0219 (10)	−0.0024 (7)	−0.0048 (8)	−0.0023 (8)
C8	0.0280 (10)	0.0100 (8)	0.0225 (10)	0.0003 (7)	−0.0009 (8)	−0.0002 (7)
C9	0.0225 (10)	0.0125 (8)	0.0164 (9)	0.0002 (7)	0.0017 (8)	−0.0001 (7)
C10	0.0210 (9)	0.0117 (8)	0.0177 (9)	0.0007 (7)	−0.0011 (8)	−0.0007 (7)
C11	0.0199 (9)	0.0132 (8)	0.0167 (9)	0.0020 (7)	−0.0011 (8)	0.0024 (7)
C12	0.0195 (9)	0.0141 (9)	0.0162 (9)	−0.0010 (7)	−0.0022 (7)	−0.0023 (7)
C13	0.0228 (10)	0.0122 (8)	0.0172 (9)	−0.0003 (7)	−0.0008 (8)	−0.0010 (7)
C14	0.0279 (11)	0.0258 (10)	0.0256 (11)	−0.0033 (8)	−0.0101 (9)	0.0040 (9)
C15	0.0234 (10)	0.0118 (8)	0.0201 (10)	−0.0012 (7)	−0.0023 (8)	0.0008 (7)
C16	0.0289 (11)	0.0115 (8)	0.0258 (10)	−0.0002 (7)	−0.0029 (8)	−0.0021 (8)
C17	0.0343 (11)	0.0147 (9)	0.0262 (10)	−0.0024 (8)	0.0029 (9)	0.0020 (8)
C18	0.0259 (10)	0.0155 (9)	0.0255 (10)	0.0021 (8)	−0.0032 (9)	−0.0012 (8)
C19	0.0346 (12)	0.0218 (10)	0.0384 (13)	−0.0040 (9)	−0.0087 (11)	−0.0066 (10)
C20	0.0266 (11)	0.0205 (10)	0.0295 (11)	0.0007 (8)	−0.0121 (9)	−0.0006 (8)

### Geometric parameters (Å, °)

**Table:**

O1—C12	1.364 (2)	C8—H8A	0.9300
O1—C11	1.368 (2)	C9—C10	1.445 (2)
O2—C9	1.259 (2)	C9—C13	1.451 (2)
O3—C1	1.356 (2)	C10—C11	1.409 (2)
O3—H1O3	0.94 (3)	C12—C13	1.397 (2)
O4—C3	1.352 (2)	C14—H14A	0.9600
O4—C14	1.424 (2)	C14—H14B	0.9600
O5—C5	1.359 (2)	C14—H14C	0.9600
O5—H1O5	0.83 (3)	C15—C18	1.520 (3)
O6—C6	1.363 (2)	C15—C17	1.535 (3)
O6—C20	1.424 (2)	C15—C16	1.551 (3)
C1—C2	1.378 (3)	C16—H16A	0.9600
C1—C10	1.413 (2)	C16—H16B	0.9600
C2—C3	1.405 (2)	C16—H16C	0.9600
C2—H2A	0.9300	C17—H17A	0.9600
C3—C4	1.409 (2)	C17—H17B	0.9600
C4—C11	1.400 (2)	C17—H17C	0.9600
C4—C15	1.558 (2)	C18—C19	1.322 (3)
C5—C6	1.392 (3)	C18—H18	0.97 (2)
C5—C12	1.393 (2)	C19—H19B	0.98 (2)
C6—C7	1.398 (2)	C19—H19A	1.00 (2)
C7—C8	1.383 (3)	C20—H20A	0.9600
C7—H7A	0.9300	C20—H20B	0.9600
C8—C13	1.396 (3)	C20—H20C	0.9600
			
C12—O1—C11	120.87 (13)	C5—C12—C13	121.75 (16)
C1—O3—H1O3	99.5 (16)	C8—C13—C12	118.73 (16)
C3—O4—C14	120.28 (15)	C8—C13—C9	123.04 (16)
C5—O5—H1O5	106.1 (16)	C12—C13—C9	118.23 (16)
C6—O6—C20	118.38 (13)	O4—C14—H14A	109.5
O3—C1—C2	118.90 (16)	O4—C14—H14B	109.5
O3—C1—C10	120.77 (16)	H14A—C14—H14B	109.5
C2—C1—C10	120.32 (16)	O4—C14—H14C	109.5
C1—C2—C3	119.70 (17)	H14A—C14—H14C	109.5
C1—C2—H2A	120.2	H14B—C14—H14C	109.5
C3—C2—H2A	120.2	C18—C15—C17	112.15 (15)
O4—C3—C2	120.79 (16)	C18—C15—C16	102.82 (14)
O4—C3—C4	116.07 (15)	C17—C15—C16	109.42 (15)
C2—C3—C4	123.13 (17)	C18—C15—C4	112.47 (15)
C11—C4—C3	114.54 (16)	C17—C15—C4	109.27 (14)
C11—C4—C15	121.41 (15)	C16—C15—C4	110.54 (14)
C3—C4—C15	124.04 (16)	C15—C16—H16A	109.5
O5—C5—C6	123.24 (16)	C15—C16—H16B	109.5
O5—C5—C12	118.53 (16)	H16A—C16—H16B	109.5
C6—C5—C12	118.23 (16)	C15—C16—H16C	109.5
O6—C6—C5	114.41 (15)	H16A—C16—H16C	109.5
O6—C6—C7	124.65 (16)	H16B—C16—H16C	109.5
C5—C6—C7	120.93 (17)	C15—C17—H17A	109.5
C8—C7—C6	119.81 (17)	C15—C17—H17B	109.5
C8—C7—H7A	120.1	H17A—C17—H17B	109.5
C6—C7—H7A	120.1	C15—C17—H17C	109.5
C7—C8—C13	120.51 (16)	H17A—C17—H17C	109.5
C7—C8—H8A	119.7	H17B—C17—H17C	109.5
C13—C8—H8A	119.7	C19—C18—C15	126.7 (2)
O2—C9—C10	121.10 (16)	C19—C18—H18	119.3 (12)
O2—C9—C13	122.15 (16)	C15—C18—H18	113.4 (12)
C10—C9—C13	116.75 (15)	C18—C19—H19B	120.2 (13)
C11—C10—C1	117.58 (16)	C18—C19—H19A	122.6 (13)
C11—C10—C9	121.29 (16)	H19B—C19—H19A	116.8 (18)
C1—C10—C9	121.10 (15)	O6—C20—H20A	109.5
O1—C11—C4	116.11 (15)	O6—C20—H20B	109.5
O1—C11—C10	119.40 (15)	H20A—C20—H20B	109.5
C4—C11—C10	124.49 (16)	O6—C20—H20C	109.5
O1—C12—C5	115.05 (15)	H20A—C20—H20C	109.5
O1—C12—C13	123.19 (16)	H20B—C20—H20C	109.5
			
O3—C1—C2—C3	179.39 (16)	C3—C4—C11—C10	−5.7 (3)
C10—C1—C2—C3	−2.0 (3)	C15—C4—C11—C10	175.57 (17)
C14—O4—C3—C2	3.8 (3)	C1—C10—C11—O1	−176.47 (15)
C14—O4—C3—C4	−177.21 (16)	C9—C10—C11—O1	5.1 (3)
C1—C2—C3—O4	177.55 (16)	C1—C10—C11—C4	2.8 (3)
C1—C2—C3—C4	−1.4 (3)	C9—C10—C11—C4	−175.67 (17)
O4—C3—C4—C11	−174.00 (15)	C11—O1—C12—C5	−177.07 (15)
C2—C3—C4—C11	5.0 (3)	C11—O1—C12—C13	2.6 (2)
O4—C3—C4—C15	4.7 (3)	O5—C5—C12—O1	−2.9 (2)
C2—C3—C4—C15	−176.34 (17)	C6—C5—C12—O1	177.22 (15)
C20—O6—C6—C5	173.27 (16)	O5—C5—C12—C13	177.41 (16)
C20—O6—C6—C7	−7.4 (3)	C6—C5—C12—C13	−2.5 (3)
O5—C5—C6—O6	1.3 (3)	C7—C8—C13—C12	0.6 (3)
C12—C5—C6—O6	−178.80 (15)	C7—C8—C13—C9	−179.37 (17)
O5—C5—C6—C7	−178.04 (17)	O1—C12—C13—C8	−178.41 (16)
C12—C5—C6—C7	1.8 (3)	C5—C12—C13—C8	1.3 (3)
O6—C6—C7—C8	−179.31 (17)	O1—C12—C13—C9	1.6 (3)
C5—C6—C7—C8	0.0 (3)	C5—C12—C13—C9	−178.73 (16)
C6—C7—C8—C13	−1.2 (3)	O2—C9—C13—C8	−1.6 (3)
O3—C1—C10—C11	179.96 (16)	C10—C9—C13—C8	177.72 (16)
C2—C1—C10—C11	1.3 (3)	O2—C9—C13—C12	178.43 (17)
O3—C1—C10—C9	−1.6 (3)	C10—C9—C13—C12	−2.3 (2)
C2—C1—C10—C9	179.76 (17)	C11—C4—C15—C18	−160.22 (17)
O2—C9—C10—C11	178.32 (17)	C3—C4—C15—C18	21.2 (2)
C13—C9—C10—C11	−1.0 (3)	C11—C4—C15—C17	74.6 (2)
O2—C9—C10—C1	−0.1 (3)	C3—C4—C15—C17	−104.0 (2)
C13—C9—C10—C1	−179.36 (16)	C11—C4—C15—C16	−45.9 (2)
C12—O1—C11—C4	174.78 (15)	C3—C4—C15—C16	135.50 (18)
C12—O1—C11—C10	−5.9 (2)	C17—C15—C18—C19	−8.8 (3)
C3—C4—C11—O1	173.53 (15)	C16—C15—C18—C19	108.6 (2)
C15—C4—C11—O1	−5.2 (2)	C4—C15—C18—C19	−132.5 (2)

### Hydrogen-bond geometry (Å, °)

**Table:**

*D*—H···*A*	*D*—H	H···*A*	*D*···*A*	*D*—H···*A*
O3—H1O3···O2	0.95 (3)	1.65 (3)	2.5573 (18)	160 (2)
O5—H1O5···O6	0.83 (2)	2.25 (2)	2.7019 (19)	115 (2)
O5—H1O5···O2^i^	0.83 (2)	1.98 (2)	2.7520 (18)	155 (2)
C8—H8A···O5^ii^	0.93	2.54	3.413 (2)	157

Symmetry codes:  (i) −*x*+1, *y*−1/2, −*z*+3/2; (ii) −*x*+1, *y*+1/2, −*z*+3/2.
